# Senescent Tumor CD8^+^ T Cells: Mechanisms of Induction and Challenges to Immunotherapy

**DOI:** 10.3390/cancers12102828

**Published:** 2020-09-30

**Authors:** Wei Liu, Paweł Stachura, Haifeng C. Xu, Sanil Bhatia, Arndt Borkhardt, Philipp A. Lang, Aleksandra A. Pandyra

**Affiliations:** 1 Department of Molecular Medicine II, Medical Faculty, Heinrich-Heine-University, 40225 Düsseldorf, Germany; weliu100@uni-duesseldorf.de (W.L.); stachura@uni-duesseldorf.de (P.S.); xuh@uni-duesseldorf.de (H.C.X.); langp@uni-duesseldorf.de (P.A.L.); 2 Department of Pediatric Oncology, Hematology and Clinical Immunology, Medical Faculty, Center of Child and Adolescent Health, Heinrich-Heine-University, 40225 Düsseldorf, Germany; Sanil.Bhatia@uni-duesseldorf.de (S.B.); arndt.borkhardt@hhu.de (A.B.); 3 Department of Gastroenterology, Hepatology, and Infectious Diseases, Heinrich-Heine-University, 40225 Düsseldorf, Germany

**Keywords:** CD8^+^ T cells, senescence, immunotherapy, metabolism

## Abstract

**Simple Summary:**

Immunotherapies harness the hosts’ immune system to combat cancer and are currently used to treat many tumor types. Immunotherapies mainly target T cells, the major immune population responsible for tumor-cell killing. One of the reasons that T cells may not respond to immunotherapeutic treatment is that they are in a dysfunctional state termed senescence. This review seeks to describe the molecular mechanisms that characterize and induce T cell senescence within the context of the tumor microenvironment and how this might affect treatment responses.

**Abstract:**

The inability of tumor-infiltrating T lymphocytes to eradicate tumor cells within the tumor microenvironment (TME) is a major obstacle to successful immunotherapeutic treatments. Understanding the immunosuppressive mechanisms within the TME is paramount to overcoming these obstacles. T cell senescence is a critical dysfunctional state present in the TME that differs from T cell exhaustion currently targeted by many immunotherapies. This review focuses on the physiological, molecular, metabolic and cellular processes that drive CD8^+^ T cell senescence. Evidence showing that senescent T cells hinder immunotherapies is discussed, as are therapeutic options to reverse T cell senescence.

## 1. Introduction

Harnessing the immune system to treat solid and hematological malignancies has ushered a novel therapeutic era. The tumor micro-environment (TME) is complex, with many targeting opportunities due to the signaling networks and cross-talk between immune, tumor and stromal cells. However, the modulation of cytotoxic antigen-activated CD8^+^ T (T_c_) cells has been at the forefront of the immunotherapy revolution [1]. The antigen-specific process requires the engagement of the T cell antigen receptor (TCR)-CD3 complex on T_c_ cells with a major histocompatibility complex (MHC) class I-bound tumor antigen-derived peptide as well as co-stimulatory signals. Responsible for the direct tumor cell killing through granule exocytosis [2], T_c_ cells are integral in eradicating tumors and are currently targeted by many approved immunotherapies. The monoclonal antibody checkpoint inhibitors such as nivolumab [3] are currently used to treat solid tumors, and they target the inhibitory programmed cell death 1 (PD-1) [4] receptor expressed on T cells. In the adoptive T cell therapy field, a patient’s T cells are expanded ex vivo, transduced with synthetic chimeric antigen receptors (CAR) targeting a tumor specific target antigen such as CD19, and transferred back to lymphodepleted patients. Currently approved CAR-T cell therapies include the second generation anti-CD19 CAR T-cell products axicabtagene ciloleucel and tisagenlecleucel for the treatment of B-cell malignancies [5,6]. Many more T cell based therapies are currently in the experimental phase of pre-clinical or clinical testing [7]. While immunotherapies have made remarkable progress in increasing the survival of some patients, low response rates, toxicities, as well as lack of available bio-markers in predicting response, make the successful implementation of these therapies challenging. A major obstacle is the inability to effectively target T_c_ cells. This can occur through T_c_ intrinsic or acquired resistance helped by dysfunctional states present within the immunosuppressive networks [8] of the TME: exhaustion and senescence. While T cell exhaustion has been extensively studied and targeted, T cell senescence, especially within the context of anti-tumor immunity, is an emerging concept in the field of T cell dysfunction. This review focuses on senescence in the CD8^+^ T cell compartment. It aims to explore the different mechanisms that induce senescence in the context of TME, ways in which T cell senescence affects responses to immunotherapies and how T cell senescence can be therapeutically reversed.

## 2. Exhaustion and Senescence

Both exhausted and senescent T cells have been found to accumulate during chronic viral infections [9,10] and cancers [11,12]. Exhaustion and senescence are both considered dysfunctional states. They are characterized by dampened granzyme B (GzmB)—mediated effector function and impaired proliferation [13]. However, they are defined by distinct surface marker, cytokine, transcriptional and metabolic profiles (Table 1).

**Figure 1:**
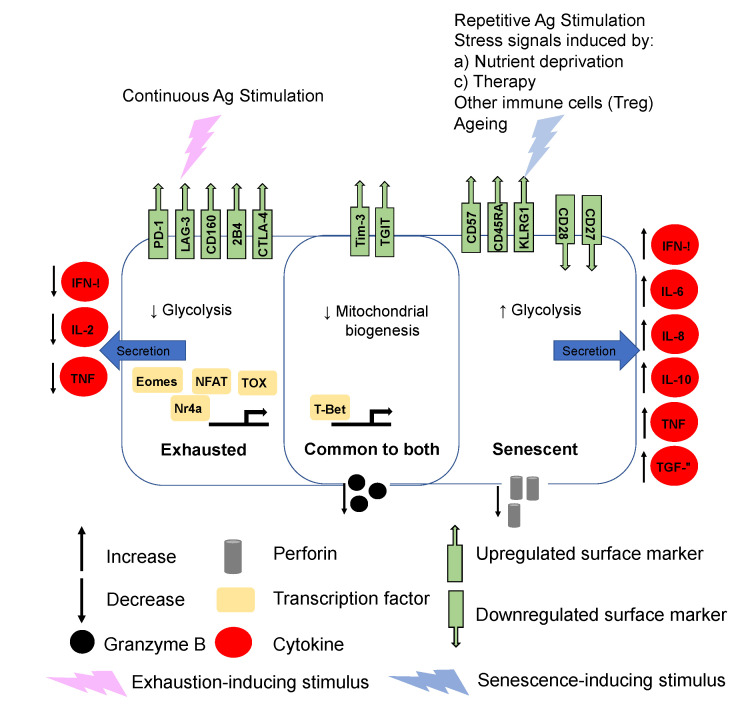
Surface phenotypic, metabolic and transcriptional differences between CD8^+^ dysfunctional senescent and exhaustive states. Characteristics common to both dysfunctional states are shown the in the middle, purple overlapping section. While both T cell state exhibit decreased effector function, senescent T cells have a very distinct senescence-associated secretory phenotype (SASP) with increased cytokine production of IFN-γ, IL-6, IL-8, IL-10, TNF and TGF-β. In contrast. exhausted T cells are characterized by decreased IL-2, TNF and IFN-γ production. While some surface markers such as Tim-3 and TGIT are common to both dysfunctional T cell states, there is otherwise quite a distinct pattern of expression. Ag = antigen.

When T_c_ cells are exhausted through excessive and continuous stimulation, they upregulate inhibitory cell surface receptors such as PD-1 and LAG-3 and possess a decreased capacity to secrete interleukin 2 (IL-2) and interferon gamma (IFN-γ) [14] (Figure 1). The exhausted transcriptional T_c_ profile is very context dependent and is driven, during varying stages of exhaustion, by nuclear factor of activated T cell (NFAT), nuclear receptor Nr4a, thymocyte selection-associated HMG box (TOX), eomesodermin (Eomes) and T-Bet [15]. While exhausted and senescent T_c_ cells share characteristics such as the upregulation of surface markers Tim-3 and tyrosine-based inhibitory motif (ITIM) domain (TIGIT), senescent T_c_ cells also upregulate CD57 and CD45RA (Figure 1). The cytokine secretory profile of senescent T_c_ cells sharply contrasts that of exhausted T cells (Figure 1). Senescent T_c_ cells secrete high levels of inflammatory cytokines such as IL-2, IL-6, IL-8, TNF, IFN-γ and the immunosuppressive IL-10 and TGF-β (Figure 1), a program known as senescence-associated secretory phenotype (SASP). This in turn has critical consequences not only for T cell themselves, but for other immune cells within the TME milieu, including antigen presenting cells (APCs) such as dendritic cells (DCs), tumor-associated macrophages (TAMs) and myeloid-derived suppressor cells (MDSC). Transcriptional programs in senescent T_c_s have been shown to be mediated by T-bet [16] but otherwise are poorly characterized. Senescence-inducing stimuli include exposure to DNA damaging agents, stress signals and repetitive stimulation linked but not limited to the ageing process. It should be pointed out that dysfunctional T cell states other than exhaustion and senescence such as anergy have also been described. Anergic T cells are hypo-responsive, produce low levels of IL-2 and generally have little effector function. T cell anergy is caused by insufficient CD28 dependent co-stimulation of the TCR, but the surface markers of anergic T cells are poorly characterized [17]. Insufficient CD28 stimulation within the TME combined with tumor cell expressing factors, such as PD-L1 and CD95, is also closely linked to deletion of effector T cells through a process known as tolerance. Tolerance is exacerbated by TGF-β and IL-10 [18]. Taken together, senescent, anergic and exhausted T_c_ cells often co-exist in the TME or circulation and simultaneously exert immunosuppressive effects [11,19]. The current limitations of check-point inhibitors suggest that targeting multiple dysfunctional T_c_ cell states would be therapeutically beneficial. A deeper understanding of the mechanisms and functional consequences of T cell senescence are urgently needed.

## 3. Mechanisms of T Cell Senescence Induction

**Figure 2:**
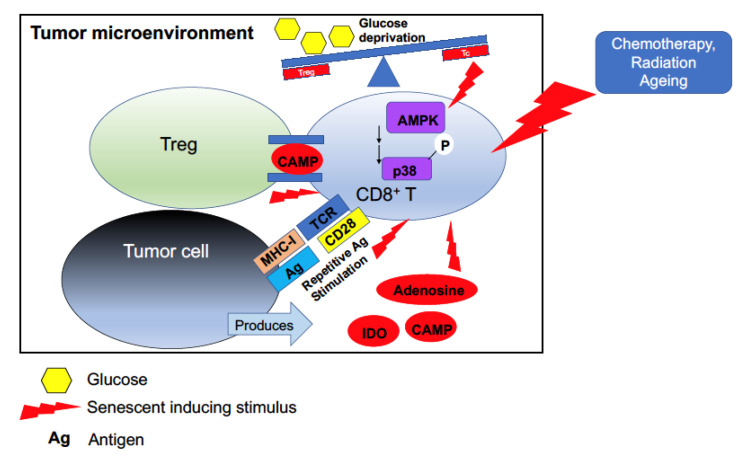
T cell senescence can occur via multiple mechanisms within the tumor microenvironment. Tregs, through glucose metabolic competition and transfer of cAMP produced by tumors, can induce CD8^+^ T cell senescence, as can other metabolites produced by tumor cells, such as adenosine. Repeated antigen stimulation and external factors such as chemotherapeutic and radiation therapy also induce premature senescence. A key molecular pathway involved in CD8^+^ T cell senescence induction is non-canonical signaling through p38-MAPK.

Senescent T cells have been found in primary and metastatic solid tumor sites [19,20,21,22,23] as well as hematological malignancies [11,24]. T_c_ senescence can be classified into two major cellular mechanisms which are however not entirely separated from one another: premature and replicative senescence. Premature senescence is caused by external factors such as stress within the TME incurred by (i) effects of immune and tumor cells, (ii) TME metabolic changes, (iii) and drug and radiation therapy, all of which are closely interlinked and not necessarily independently occurring events (Figure 2). Replicative senescence is linked to age-related changes and to telomeric shortening. T cell anergy on the other hand, is closely linked to peripheral tolerance.

### 3.1. Signaling Pathways Involved in T_c_ Senescence

The intrinsic molecular pathways governing premature and replicative senescence are not completely defined but involve the MAPK pathway. The diverse and complex MAPK pathway with its three subgroups, Erk, Jnk and p38, is involved in many aspects of innate and acquired immune regulation. Classical engagement of the MAPK pathway downstream of the TCR does not occur in senescent T_c_ cells as they lack the costimulatory molecule CD28. p38-MAPK activation however, can also be mediated by environmental stressors such as low glucose, DNA damage and by proinflammatory cytokines which trigger AMPK to auto-phosphorylate p38. Henson et al. showed that p38 MAPK expression was elevated in human senescent CD8^+^ T cells and that blocking p38 following inhibitor treatment resulted in increased mitochondrial mass, improved mitochondrial function and enhanced proliferation [25]. Human senescent CD27^–^CD28^–^CD4^+^ T cells prompt AMPK-stimulated recruitment of p38, resulting in p38 autophosphorylation, facilitated by the protein scaffold TAB1, which inhibits telomerase activity and parts of the TCR signalosome [26]. In addition to p38, involvement of the other MAPK members Erk and Jnk have been found to impact T cell senescence in the context of a new immune inhibitory complex termed the sestrin—MAPK activation complex (sMAC). sMAC formation is increased with age [27]. In human and murine CD4^+^ T cells, sestrins induced senescence through binding and simultaneous activation of Erk, Jnk and p38 to form sMAC. Silencing sestrin enhanced T cell activity. Although CD4^+^ T cells were experimentally used in this study, the authors did show that sestrin deficiency increased vaccine responses in aged mice and increased the frequency of splenic CD8^+^ T cells. A follow-up study specifically examining the effects of sestrin in CD27^−^CD28^−^CD8^+^ T cells, showed that a divergent mechanism was responsible for sestrin-dependent senescence in CD27^−^CD28^−^CD8^+^ T cells linked to a natural killer group 2 member D (NKG2D)–DNAX Activating Protein of 12KDa (DAP12)–sestrin 2 complex. Disturbance of the NKG2D–DAP12–sestrin 2 complex through sestrin 2 genetic inhibition, restored TCR signaling [28]. T cell mitochondrial dysfunction also accelerates senescence. It was recently demonstrated that *Tfam^fl/fl^ Cd4^Cre^* mice (where mitochondrial transcription factor A (TFAM) is depleted in CD4^+^ and CD8^+^ lymphocytes) prematurely died due to multiple age-related changes [29]. Taken together, the full extent of the molecular pathways involved T_c_ senescence are not completely elucidated. The current knowledge, however, presents targetable opportunities to potentially reverse senescence and understand how senescent T_c_ cells might impact immunotherapies in the treatment of cancer.

### 3.2. The TME Drives Tc Premature Senescence

#### 3.2.1. Immune and Tumor Cells

A tumor’s ability to evade the immune system is dynamic, complex and partially dependent on the immunosuppressive activities of infiltrating immune cells. T_c_ effector function is similarly complicated and shaped by the spatiotemporal distribution of APCs in the tumor milieu and tumor-draining lymph nodes, cytokines and the presence of other immune cells such as regulatory CD4^+^CD25^hi^FoxP3^+^ T (Treg) cells. Initial priming of naïve T cells occurs in the lymph node through direct interaction with antigen present on APCs such as DCs. DCs also co-express receptors such as CD80 necessary for binding to CD28 and inducing co-stimulatory signals. Upon migration of primed T_c_ cells into the TME, the tumor cells expressing the antigenic peptide become targets. The numbers of infiltrating CD8^+^ T cells varies widely across tumor types. Some tumors, such as melanoma and non-small cell lung cancer (NSCLC), generally have a high degree T_c_ infiltration. Other tumors, such as pancreatic and neuroblastoma, typically have a low degree of T_c_ infiltrates, although of course within a specific tumor type, there is a lot of intra-tumoral heterogeneity [30]. Many factors during this process can impact the ability of T_c_ cells to target tumor cells.

Regulatory CD4^+^CD25^hi^FoxP3^+^ T (Treg) cells are subsets of T cells which play a role in maintaining immune homeostasis and present a critical barrier for immunotherapies through their suppressive effects on T_c_ cells. Tregs have been found in lymph nodes where they impact DC function through CCL22. CCL22, a chemokine produced by dendritic cells, enables cell-to-cell contact between DCs and Treg through Treg-expressed CCR4 [31]. Tregs accumulate within the TME, and their ability to infiltrate into tumors has been linked to the expression of multiple chemokine receptors such as CCR4, CCR5, CCR8 and CCR10. Within the TME, Tregs usually express immunosuppressive molecules such as CTL-4, which binds to CD80 and CD86 on APCs thereby affecting T_c_ effector function [32]. Treg suppressive mediated-effects on APCs and T_c_ effector cells can also occur through inhibitory cytokine secretion of IL-10, TGF-β, and IL-35. These inhibitory cytokines suppress antigen presentation in APCs. IL-35 and IL-10 promote T cell exhaustion. Metabolic competition for the consumption of IL-2 through the expression of CD25 on Tregs also suppresses T_c_ effector functions [33]. Tregs are also found in peripheral circulation, but their precise role in facilitating immune evasion are not as well characterized as with the TME-associated Tregs [32].

Relating specifically to Treg-mediated T_c_ senescence induction, an important study demonstrated that co-transfer of Tregs and naïve CD8^+^ T cells into *Rag1^−/−^* mice transformed naïve T cells into senescent T cells (as assessed by SA-β-Gal positivity). Furthermore, the senescent T cells acquired immunosuppressive functions both in vitro and in vivo. Involvement of the mitogen-activated protein kinase (MAPK) pathway was implicated, as pre-treatment with ERK and p38 inhibitors abrogated these immunosuppressive effects [34]. Another critical study by Liu at al. using ex vivo cultured primary human T cells demonstrated that human Tregs induced nuclear kinase ataxia–telangiectasia-mutated protein (ATM)-associated DNA damage responses in T_c_s [35]. The majority of subsequent mechanistic experiments demonstrated that senescence was mediated by competition for glucose, which triggered phosphorylation of the energy sensor AMP-activated protein kinase (AMPK) in cooperation with Stat1 and Stat3. These mechanistic studies were performed using naïve CD4^+^ T cells. However, the authors did show that Treg-induced naïve CD8^+^ T cell senescence was blocked by pre-treatment with ATM or STAT inhibitors in NOD SCID gamma (NSG) mice, indicating that similar mechanisms of senescence induction also occur in CD8^+^ T cells. These studies did not directly explore the specific co-localized induction of Treg-induced T_c_ senescence within the TME. However, as Tregs and T_c_s are often co-expressed within tumors [36,37] and tumor-draining lymph nodes [38], the clinical relevance of these in vivo and ex vivo studies is plausible. These interactions, however, would likely be mediated by APCs, which these studies did not address. APCs might guide the cellular interaction between Tregs and T_c_s through costimulatory (CD28/CD80 and CD86, OXO/OXOL, CD95/CD95L) on CD8^+^ T cells or co-inhibitory (PD-1/PD-L1 and CTLA-4/CD80) signals on CD8^+^ and CD4^+^ T cells, respectively. Other immune cells within the TME might also indirectly impact T_c_ senescence. Treg and MDSC populations often support each other’s expansion through positive feedback loops involving TGF-β and other cytokines [33]. In turn, MDSCs can be expanded by many of the SASP cytokines secreted by T_c_ senescent cells such as TGF-β, IL-6 and IFN-γ [39].

Tumor cells are the targets of T_c_s following priming in the lymph nodes. T_c_-mediated tumor cell killing occurs through TCR-mediated T_c_ cell binding to MHC-I-restricted tumor antigens on tumor cells. This interaction releases cytolytic perforin and granzymes causing tumor cell killing. Tumor cell killing can also occur through the death receptor pathway mediated by the expression of CD95 on tumor cells and CD95L on T_c_ cells. Receptors present on tumor cells such as PD-L1 can diminish T_c_ cells responses through the PD-1–PD–L1 axis [2]. Tumor cells can also downregulate MHC-I and CD95 expression to dampen responses and evade apoptosis. Immunosuppressive cytokines produced by tumor cells such as TGF-β and IL-10 also influence T_c_ effector function. Metabolites produced by tumor cells, elaborated on in the section below, can also affect T_c_s.

Tumor cells have been shown to induce T_c_ senescence in in vitro and in vivo models. Montes et al. showed that ex vivo incubation of human T cells isolated from healthy donors with a variety of human tumor cell lines triggered downregulation of CD28 expression. Activation of ATM, shortening of telomere length and an ability to suppress antigen nonspecific and allogeneic-induced proliferation of responder T cells were also observed [40]. Tumor-induced senescence was dependent on direct tumor-T cell contact [40]. Another pivotal investigation demonstrated that adoptive transfer of tumor-specific CD8^+^ tumor-infiltrating lymphocytes (TIL) 586 cells into tumor-bearing (586 mel cells) NSG mice induced senescence, as assessed by SA-β-Gal^+^ staining in TILs [41]. Activation of TLR8 signaling in tumor cells was able to reverse the tumor-induced senescence [42]. Taken together, tumor-infiltrating immune cells such as Tregs as well as tumor cells have been shown to be capable of inducing T cell senescence. As this evidence largely stems from ex vivo studies, the role of critical cells mediating T_c_ priming such as DCs in facilitating this process remains to be explored.

#### 3.2.2. Metabolic Changes

Metabolic re-programming within the TME is critical to many pro-tumorigenic processes, including driving senescence [43,44]. Cancer cells have a high rate of glucose consumption through aerobic glycolysis, resulting in low glucose and high lactate concentrations in the TME [45]. Antigen-activated effector T cells, once they become primed and activated in the lymph nodes, begin their clonal expansion and rapid proliferation [43]. Therefore, they have metabolic requirements different to those of circulating naïve cells which rely on oxidative phosphorylation for their energy requirements. Rapidly proliferating T cells have higher glycolytic activity [46] and increased amino acid metabolism [47]. TCR-mediated T cell activation is followed by metabolic re-programming and biomass accumulation. Changes in metabolism include a switch to aerobic glycolysis despite there being enough oxygen present to generate glucose through the tricarboxylic acid (TCA) cycle [43]. Aerobic glycolysis provides important intermediates for cell growth, such as glucose-6-phosphate, 3-phosphoglycerate (3PG) and citrate. Molecularly, this metabolic transition is supported by mTOR, PI3K activity, the transcription factor Myc and hypoxia-inducible factor-1α (HIF-1α) [43,47]. As already described above in the study by Liu et al., increased glucose consumption by Tregs reduced the glucose pool available for naïve T cells, initiating AMPK signaling cascades and DNA damage responses [35]. Effector T cell activity is sensitive towards intracellular NAD depletion, often occurring in the TME. Tregs, however, have developed re-programming strategies mediated by the transcription factor FOXP3 to maintain their proliferative capabilities and suppressive functions, despite low glucose and high lactate levels [48]. It remains to be elucidated whether within the TME Treg numbers would be high enough to deplete glucose pools. However, in addition to Treg competing for glucose consumption, other immune cells within the TME also have distinct metabolic requirements which can affect glucose pools. MDSCs have high glucose uptake rates and can contribute to the dysfunction of other immune cells by limiting pools of available glucose [39]. MDSCs can also affect the T cell activation through depletion of amino acids such as cystine and cysteine [49], but whether that eventually also contributes to T_c_ senescence induction is not known. Conditions of hypoxia within the TME compounded by increased tumor acidity can cause an accumulation of immunosuppressive M2 polarized TAMs, which are critical in maintaining a tolerogenic phenotype, expanding Tregs and suppressing T_c_ function [50].

Tumor cells also produce metabolites that are inducers of T_c_ cell senescence. For instance adenosine, whose production is catalyzed by the surface ectonucleotidases CD39 and CD73, accumulates in the TME through CD38 and CD73 expression on cancer exosomes [51]. Adenosine exposure triggered replicative senescence in human CD8^+^ T cells, decreased proliferative capacity and reduced IL-2 production [52]. Furthermore, adenosine can impact APCs that are critical for T_c_ function. Tumor-produced adenosine has been shown to decrease DC maturation and immune function [53]. Another example is cyclic adenosine monophosphate (cAMP), produced by tumor cells and a suppressor of T cell function [54]. In ex vivo co-culturing experiments, cAMP was shown to be transferred from Tregs to T_c_s through direct gap junction formation, thereby suppressing proliferation of T_c_s and decreasing IL-2 production [55]. However, the in vivo relevance of these findings in the context of the TME has yet to be validated. Other TME-associated metabolites such as indoleamine 2,3-dioxygenase (IDO) [56], although not yet shown to directly induce T_c_ senescence, plausibly contribute to the induction of T_c_ dysfunction through activation of Tregs.

#### 3.2.3. Chemotherapeutics and Radiation Therapy

DNA damage caused by commonly used chemotherapeutics can lead to senescence induction in both tumor and normal cells [57]. Most chemotherapeutic agents are genotoxic and cause DNA damage by triggering chromosomal breaks or double stranded DNA breaks. This is followed by induction of the DNA damage response (DDR) mediated by ATM and ATR kinases, whose downstream targets are cell cycle regulatory proteins checkpoint homologs 1 and 2 (Chk1 and Chk2). Chk1 and Chk2 trigger the activation of various cyclin-dependent kinase inhibitors causing cell cycle arrest [58]. It is not surprising that treatment with such agents can also lead to immuno-senescence, especially in rapidly proliferating populations such as T_c_ cells following antigen exposure. A six months longitudinal study tracked shifts in CD8^+^ T cell populations in DNA-damaging chemotherapy-treated breast cancer patients. The study found that senescent-enriched CD28^−^CD57^+^ cells were more pre-dominant in cancer patients compared to the untreated healthy age-matched group. The study also found that immuno-senescence and immune risk parameters were more pronounced in the chemotherapy-treated group [22]. When peripheral CD8^+^ T lymphocytes were assessed in metastatic breast cancer patients during the post-salvage taxane chemotherapy follow-up, it was found that CD8^+^CD28^−^ populations were increased in breast cancer patients compared to the control cohort [23]. In another longitudinal study, radiotherapy and chemotherapy in early-stage breast cancer patients increased senescent cytotoxic T lymphocytes [59]. Shortened telomere length was observed in peripheral blood mononuclear cells in non-Hodgkin’s lymphoma patients undergoing chemotherapy [60]. These results are correlative, and the functional consequences of T_c_ cell senescence induction in these clinical settings should be mechanistically explored. Tumor senescence might be beneficial to the organism under some contexts, as it stops tumor-cell proliferation. However, the unintended potentially detrimental consequences of T_c_ cell senescence should be considered during the course of therapy, especially if immunotherapy treatment such as CAR-T cell therapy is to be further applied.

### 3.3. Age-Related Replicative Senescence

Replicative senescence in normal somatic cells is telomere dependent and occurs as a result of telomere shortening or a classical DNA damage response triggered by a dysfunctional telomerase [61]. Telomerase dysfunction can be triggered by oxidative stress [62]. This is paralleled in T_c_ cells. The natural aging process is accompanied by blunted immune responses to anti-viral, bacterial and other stimuli as well as decreased responsiveness to vaccines [63,64]. Repeated antigen stimulation throughout an individual’s life time is one potential cause of aging-induced T_c_ senescence. Others include physiological changes such as thymic shrinking which limits the naïve T cell pool, changes in the bone marrow, and obesity [62,65,66]. While ex vivo stimulated human CD8^+^ T cells have been shown to have high initial telomerase activity, their telomere lengths shorten following several rounds of antigen stimulation, and their telomerase activity dramatically decreases [67]. A decrease in the number of naïve CD8^+^ T cells and suppressed functionality in terms proliferation and decreased cytokine production accompany age-related dysfunction [63,68]. In aged mice, proliferative defects have also been demonstrated in anti-viral memory CD8^+^ T cells [69]. Senescence has also been observed in CD8^+^ T cells which have not undergone repetitive antigen stimulation. A recent study explored a specialized subset of semi-differentiated antigen naïve but semi-primed T cells expressing the activation marker CD44 termed “virtual memory” CD8^+^ T cells (CD44^hi^CD49d^lo^; T_VM_). The investigation found T_VM_ cells accumulated in aged mice and humans and acquired a dysfunctional senescent but not exhausted phenotype [70]. The presence of T_VM_ cells in aged individuals would be expected to diminish primary CD8^+^ T cell responses while still maintaining a base effector function and the ability to secrete cytokines.

## 4. Tc Cell Senescence and Effects on Immunotherapy Response

### 4.1. Checkpoint Inhibitors

Given the increased accumulation of senescent T_c_ cells in older individuals, it would be reasonable to expect that age would dimmish response to immunotherapies. Curiously, however, some reports indicate that advanced age positively correlates with anti-PD-1 therapy response [71]. In this study, when a total of 538 metastatic melanoma patients treated with pembrolizumab were stratified according to age and response, a smaller percentage of patients aged over 62 years had progressive disease. The study, which controlled for prior MAPK inhibitor therapy, did not control for mutational burden but did corroborate the patient data with murine models. Genetically identical tumors experienced better, albeit minor, anti-PD-1 responses in aged mice. The study found that in younger patients, Tregs were increased and CD8^+^ T cells decreased in the TME. The authors speculated that memory CD8^+^ T cells, which accumulate with age and expand in response to immunotherapy, may be responsible for improved anti-PD-1 responses. However, accumulation of memory CD8^+^ T cells in aged individuals is not absolute and is relative to a decrease in naïve T cells due to thymic shrinking. Other studies in various tumor types have found no correlation [72,73], or a negative correlation between age and checkpoint inhibitor response [74]. The study citing the negative correlation was carried out in patients with advanced renal cell carcinoma with a small sub-group of older patients. Furthermore, the study did not assess infiltrates in the TME. Age-induced senescence might affect other critical immune cells which could impact responses to checkpoint inhibitors, including the abovementioned T_vM_ cells [70]. The phagocytic function of macrophages/monocytes is decreased during aging and might impair release of antigens into the micro-environment. Aging also decreases numbers and antigen presenting functions of APCs, which would impact T cell effector function [75].

It is difficult to conclusively ascertain whether older patients fare better/worse following immunotherapy treatment given the usually low numbers of elderly patients included in clinical trials. Immunotherapy response, particularly to checkpoint inhibitors, is complex, and no successful biomarker of response has been established. Several biomarkers have been suggested, including neo-antigen burden, PD-L1 expression, genomic and transcriptomic signatures and immune infiltration [76,77]. While age-induced T_c_ cell senescence might impact response to immunotherapy treatment, other more significant factors could over-ride these effects. For example, a recent study reported that tumors in younger female individuals accumulated more poorly presented driver mutations than those in older and male patients. Accordingly, these female patients had poorer immune checkpoint therapy responses [78]. Taken together, it remains to be proven whether age is a predictor of immunotherapy response

Irrespective of age, patients with accumulated senescent T_c_ cells within the TME might respond poorly to checkpoint inhibition, which seeks to de-repress the exhaustive T_c_ phenotype but does not target the senescent one. A small pilot study of melanoma patients was able to identify patients with primary resistance to checkpoint inhibitors by using lymphocyte phenotyping for senescence markers CD27, CD28, Tim-3 and CD57 [79]. The study tracked senescence markers in the peripheral blood for 12 weeks post diagnosis of metastatic melanoma. Another study found that in multiple myeloma, T-cell clones exhibited hypo-responsiveness and a telomere-independent senescent (KLRG-1^+^/CD57^+^/CD160^+^/CD28^−^) phenotype. These senescent T cells also expressed low levels of PD-1 and CTL-4 [24]. Although limited in the numbers of assessed patients, these studies suggest that senescent T_c_ impede responses to checkpoint inhibitors.

### 4.2. CAR-T Cell Therapy

CAR-T cell therapy has the potential to be curative in patients with hematological malignancies such as leukemia and lymphoma. However, CAR-T cell therapy application to solid tumors whose targetable antigen repertoire can be difficult to predict will be more challenging. CAR-T cell therapy depends on the isolation and expansion of a patient’s T cells harvested from the periphery. It is plausible to speculate that functionally impaired senescent T cells in this context would provide an impediment to successful T cell expansion and/or CAR activity once in the TME. In vivo, murine studies have shown that PD-1 upregulation within the tumor microenvironment impeded the function of CD28-CAR-T cells, which was restored by concomitant treatment with anti-PD-1 antibodies [80]. Furthermore, introduction of a patient’s expanded T cells into the TME might lead to terminal differentiation of the T cells caused by TME-induced T_c_ senescence or exhaustion [81]. Taken together, although this requires further exploration, there is reasonable correlative evidence to suggest that senescent T_c_ cells impact immunotherapy responses.

### 4.3. Targeting T Cell Senescence

As 40–85 percent of patients treated with checkpoint inhibitors fail to exhibit a sustained clinical response [82], combinatorial approaches that also reverse T_c_ cell senescence could be of therapeutic benefit. As induction of tumor cell senescence can be advantageous in the context of tumor clearance, strategies to reverse T cell senescence should be carefully considered. While induction of tumor cell senescence can initially stop uncontrolled proliferation, the SASP profile of senescent tumor cells can promote tumor relapse, inflammation and recruit immunosuppressive immune cells to the TME. Agents that induce apoptosis in senescent cells termed “senolytics” (e.g., dasatanib and quercetin), are currently being tested in pulmonary fibrosis and after radiotherapy to improve clinical symptoms [83,84]. Whether these agents can be used in neoplastic malignancies to clear senescent tumor cells and/or senescent T_c_ cells is an area warranting further exploration.

Agents such as ralimetinib that target p38 MAPK, which are safe and have already being used in clinical trials [85], are an attractive option as they could dually target T_c_ senescence signaling and tumor proliferation. In addition to their direct anti-tumoral effects, other inhibitors of the MAPK pathway already approved for the treatment of metastatic melanoma are also promising. These include the B-Raf-targeting inhibitor vemurafenib or MEK1/2 targeting inhibitor trametinib. These drugs have already been shown to increase the number of CD8^+^ TILs and enhance checkpoint inhibition in murine models [86,87]. During senescence, upregulation of BCL-2 family members (BCL-XL, BCL-W) have been reported in several studies, across various cell types [84]. Of note, inhibitors targeting the BCL-2 protein family, including novitoclax, are selectively senolytic in some cell types [88]. Moreover, several other pro-survival pathways have been implicated in eliminating senescence, including the p53/p21 axis, receptor tyrosine kinase, HIF-1α and serpine anti-apoptotic pathways [84,89]. HSP90, a member of the chaperone protein family, was identified as a new class of senolytics [90,91]. HSP90 is upregulated in several tumor types and promotes the stabilization of PI3K/Akt, ERK and other pro-survival signaling pathways upregulated during cellular senescence [92]. Therefore, downregulation of pro-survival signaling pathways upon HSP90 inhibition may be responsible for its senolytic activity [90]. Whether these inhibitors also reverse T_c_ senescence and enhance checkpoint inhibition in the clinical setting remains to be elucidated. However, combinatorial therapy is paramount in achieving clinical success. Therefore, these strategies might present good therapeutic opportunities. They also have the advantage of using already approved therapies.

Combining checkpoint inhibitors with other activators of the immune system such as TLR8 agonists could maximize the benefits of immunotherapy. TLR8 agonists have already been shown to reverse the T cell tumor-induced senescence in mouse models of cancer [41]. They have the added benefit of increasing immune infiltration and activating other anti-tumoral immune cells such as dendritic and NK cells. The TLR8 agonist motolimod (VTX-2337) has been evaluated in clinical trials, is well tolerated and shows promise activating the immune system in cancer patients [93,94]. Other approaches exist, such as the reprogramming of senescent T_c_ cells from pluripotent stem cells (T-IPSCs). However, this approach is complicated by the unpredictable re-arrangement of the TCR [81].

Taken together, as our molecular understanding of the pathways governing T_c_ cell senescence increases, so will the ability to effectively target this dysfunctional subset of T cells, reverse their immunosuppression and augment currently used immunotherapies. Furthermore, assessing senescent T cell accumulation following treatment with currently used therapies in cancer patients might help to optimize treatment strategies and uncover novel bio-markers of immunotherapy response.

## 5. Conclusions

Senescent T_c_ cells are phenotypically distinct from exhausted T_c_ cells. Their immunosuppressive function brings new obstacles to successful immunotherapy. Regardless of whether the tumor-specific T cell senescence is of replicative or premature origin, a deeper molecular understating of the molecular pathways driving this process is needed. This will open new therapeutic options to eradicate challenges imposed by a suppressive TME. Currently, MAPK pathway inhibitors and TLR8a agonists are amongst the most clinically promising candidates to reverse T cell senescence. They can potentially be used in combinatorial approaches with checkpoint inhibitors to target many levels of immune dysfunction and maximize anti-tumor immunity. Much progress has been made in defining T cell senescence as a distinct dysfunctional state. However, clinical evidence showing the functional importance of T cell senescence in solid tumors and hematological malignancies is largely correlative and needs to be further explored.

## Figures and Tables

**Table 1:** Characteristics of senescent and exhausted T cells.

	Senescent T Cells	Exhausted T Cells
Stimulus	Repetitive Ag Stimulation, Stress	Continuous Ag Stimulation
Cytokine Secretion	↑ IFN--γ, IL-6, IL-8, IL-10, TNF, TGF-β	↓ IFN-γ, IL-2, TNF
Surface Markers	↑ CD57, Tim-3, TGIT, CD45RA, KLRG1, ↓ CD28, CD27	↑ PD-1, LAG-3, CD 160, 2B4, CTLA-4, Tim-3, TGIT
Metabolism	↑ Glycolysis, ↓ Mitochondrial Biogenesis	↓ Glycolysis, ↓ Mitochondrial Biogenesis
Transcriptional	T-bet	Eomes, NFAT, TOX, T-bet, Nr4a
Effector Functions	↓ Granzyme B, ↓Perforin	↓ Granzyme B
Phenotypic Characteristic	↓ Proliferative Capacity, ↑ DNA Damage Molecules, ↑ SA-β-gal activity	↓ Proliferative Capacity, Cell Cycle Arrest

↓ Decrease, ↑ Increase.
